# Sex differences in 50 kHz call subtypes emitted during tickling-induced playful behaviour in rats

**DOI:** 10.1038/s41598-022-19362-7

**Published:** 2022-09-12

**Authors:** Emma K. L. Tivey, Jessica E. Martin, Sarah M. Brown, Vincent Bombail, Alistair B. Lawrence, Simone L. Meddle

**Affiliations:** 1 The Roslin Institute, The Royal (Dick) School of Veterinary Studies, The University of Edinburgh, Edinburgh, UK; 2 Animal and Veterinary Sciences, Scotland’s Rural College (SRUC), Edinburgh, UK

**Keywords:** Biological techniques, Zoology

## Abstract

‘Tickling’ induces positive affective states in laboratory rats as evidenced by the production of 50-kHz ultrasonic vocalisations (USVs), although this has mostly been investigated in males. Juvenile rats emit distinctive 50-kHz USV subtypes. Frequency-modulated (FM) 50-kHz USVs are thought to be associated with positive affect and flat 50-kHz USVs with social communication. FM and flat USVs are produced by both sexes during tickling, but it is unclear whether these calls are produced in relation to particular play-related behaviours, and whether USV subtypes are used in a sexually dimorphic manner during tickling. We tested the hypotheses that FM USVs are associated with tickle-induced play behaviours in a sex-specific way, and that flat USVs are associated with non-play activities. Rats were allocated to one of two treatment groups: tickling (tickled, n = 16/sex) or no hand contact (control, n = 16/sex). Play behaviours (hopping, darting and hand approaches) and FM and flat USVs emitted during the testing session were quantified for each rat, with the frequency of FM and flat USVs made in anticipation of, and during, each behaviour analysed. In females, play behaviours were associated with more flat USVs than in males (before and during; *p* < 0.001), irrespective of treatment. FM USVs were paired with hopping and darting (before and during;* p *< 0.001), and in anticipation of hand approaches (*p* < 0.001) in both tickled females and males compared to controls (both sexes) suggesting that FM USVs are linked with play behaviour. The higher call rate of flat USVs paired with play behaviour in females suggests that there may be sex differences in the role of flat USVs during play. This result is evidence of sex differences in tickle-induced behaviours and has implications for our understanding of the function of different USVs in juvenile female and male rats.

Heterospecific play, or tickling, has been used for over 20 years to model positive affective states in rats^1^. 50 kHz ultrasonic vocalisations (USVs) are associated with positive affective states [e.g.^2^] and rewarding stimuli, such as rough and tumble play^3^, anticipation of food^4^ and alcohol^5^, and the euphorigenic drug, amphetamine^6^, all increase the number of 50 kHz USVs produced by rats^2^. 50 kHz USVs are also abundantly produced by rats during tickling [e.g.^7^], possibly in a graded manner^8^ which may indicate individual preference to tickling^9^. Compared to 40 studies using just male rats^8,10,27,57–62^, 6 studies to date have investigated the effect of tickling on 50 kHz USV production on female rats^10,37,63^ and 21 studies have used both female and male rats, but not all have focussed directly on investigating sex differences^10,15,64,65^. Studies which used both sexes have presented varying results, with some studies showing a difference between sexes^13–15^ whereas other studies show no sex differences^11,12^. Therefore, it is unclear whether female and male rats respond differently in their USV response to being tickled, and whether tickling induces positive affect to the same extent in female and male rats. This is important in understanding the biological significance of sex differences in USV production and in terms of tickling being recommended as a welfare intervention for rats kept for scientific experimentation^16^: tickling may not have the same effect on all rats^8^ and it has been postulated that tickling, where possible, should be adapted based on the responses of the rat to the tickling stimulus^9^. Therefore, elucidating the response of both female and male rats to tickling may allow for further refinement of the advisement of tickling to be used as an intervention to improve the welfare of laboratory rats^16^.

50 kHz USVs can be classified into distinct call subtypes; currently 14 have been described, many of which have some degree of frequency modulation^17^. Whilst the role of different frequency-modulated (FM) 50 kHz call subtypes is not yet fully explained, it is widely accepted that FM 50 kHz USVs have different associations to a type of non-frequency-modulated 50 kHz USV, referred to as ‘flat’ 50 kHz USVs^17–19^. In the present study, ‘FM’ USVs will refer to calls in the 30–90 kHz frequency range, containing short (< 15 ms) sinusoidal oscillatory motifs, those include calls in *trill, complex, multistep, trill with jumps,* according to the classification in^17^. The term ‘flat’ will refer to calls that have a nearly constant frequency in the 30–90 kHz frequency range with a mean slope between − 0.2 and 0.2 kHz/ms, those include calls in *flat* USV category according to the classification in^17^. Different frequency modulated 50 kHz USV types have been reported to be associated with positive affect in young rats^18^ and are emitted during rewarding interactions such as mating, conspecific rough-and-tumble play^18^ and tickling^1^; flat 50 kHz USVs are thought to have a social communicatory role^2,17,20,21^. As different USV subtypes are produced in different contexts, it is proposed that FM and flat 50 kHz USVs may have different behavioural functions^17^, for example, sharing specific information to conspecifics^22^.

Several studies have investigated the co-occurrence of USVs with behaviours. One study^23^ found that during a hide and seek paradigm, adult male Long-Evans hooded rats emitted 50 kHz USVs differently depending on whether the rat was in the ‘hide’ or the ‘seek’ role. In the “hide trials”, rats produced low numbers of 50 kHz USVs compared to “seek trials” where the rats called more, with flat and ‘*modulated*’ subtypes being the most frequent call subtypes suggesting a role for calls in coordinating play^23^. Different call subtypes are produced in relation to specific social behaviours during anticipation of play in juvenile male Long-Evans rats^21,24^. ’*Trill*’ USVs are associated with play-initiating nape contacts and approaching the play mate in juvenile male Long-Evans rats^22^. Together this evidence suggests that male rats couple certain call subtypes with behaviour to communicate with a conspecific during play^19^, but to date most studies have investigated the relationship between calls and behavior in males. The question of whether each sex uses call subtypes differently has not been fully addressed to our knowledge.

No studies to date have focused primarily on sex differences in the response to tickling, in particular whether rats pair 50 kHz USVs with behaviours seen during tickling in a similar way to conspecific play. Discrete behaviours such as approaches^7^ and scampers^25,26^ are observed during tickling. Approach behaviours are often used in tickling studies as a measure of reward during tickling^7,27^, while scampering is a solitary play behaviour, comprised of hopping and darting^25–27^. We aimed to test the hypothesis that FM 50 kHz USVs are associated with tickled-induced play behaviours in a sex-specific way, and that flat 50 kHz USVs are associated with non-play activities in both female and male rats. Thus, this study aimed to elaborate on the function of the two call subtypes, flat and FM 50 kHz USVs, particularly to investigate potential sex differences in the behaviours with which flat and FM 50 kHz USVs are associated.

## Results

### USV production associated with play-related behaviours

#### Hopping

**Figure 1:**
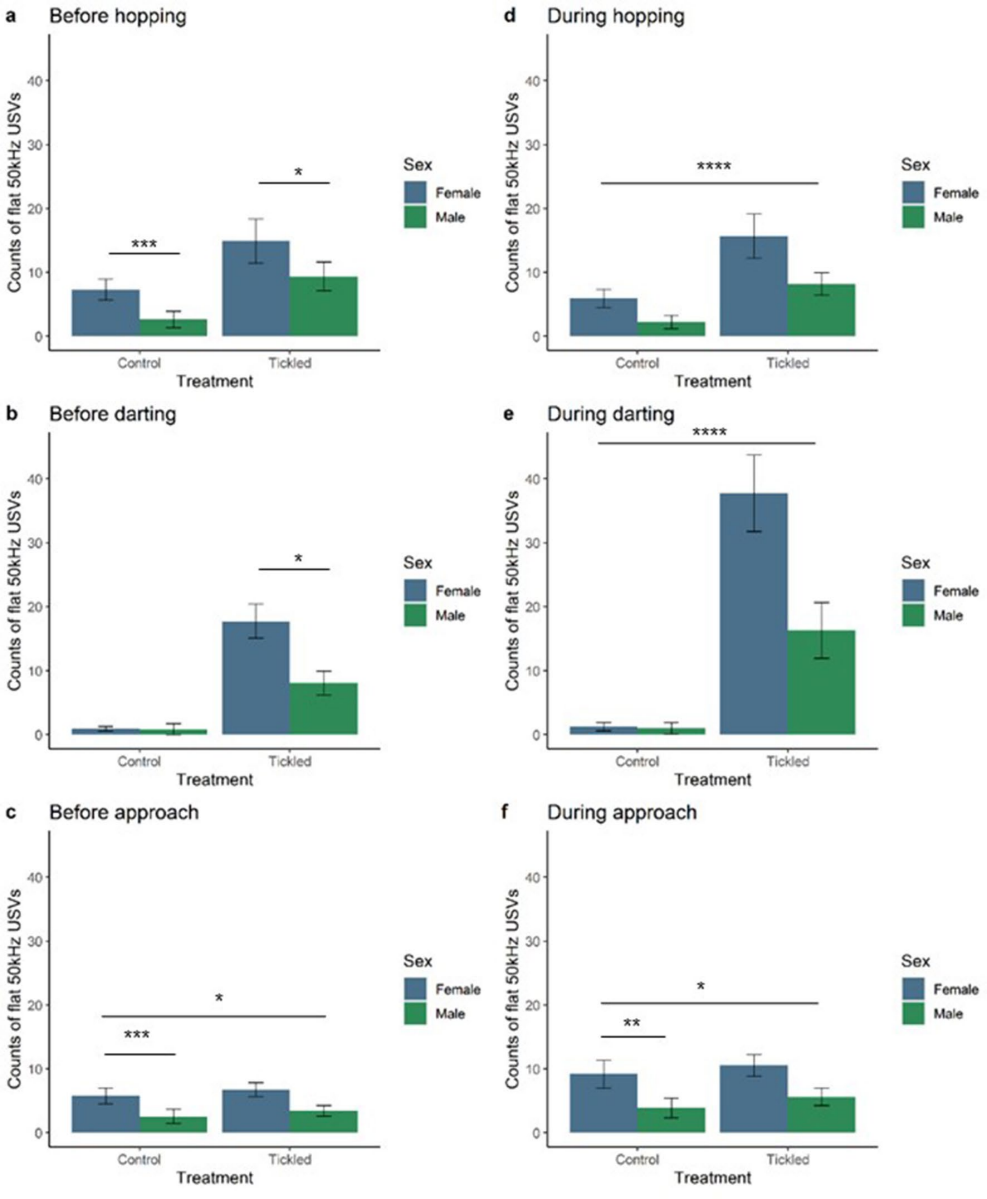
Flat ultrasonic vocalisations (USVs) paired with play-related behaviours in tickled compared to control rats. Estimated marginal means ± 95% CI of flat 50 kHz USVs produced in the one second before hopping (**a**), darting (**b**) and approach to the hand (**c**) ‘play-related’ behaviours, and flat 50 kHz USVs produced during hopping (**d**), darting (**e**) and approach to the hand (**f**) ‘play-related’ behaviours. Female (blue) and male rats (green). n = 16/group. *denotes *p* ≤ 0.05; **denotes *p* ≤ 0.01; *** denotes *p* ≤ 0.001; **** denotes *p* ≤ 0.0001 for significant pairwise interactions.

There was a sex difference in flat 50 kHz USVs when paired with hopping. Female rats emitted more flat 50 kHz USVs before (X^2^_(1,63)_ = 11.16,* p *= 0.0008), and during (X^2^_(1,63)_ = 7.78,* p *= 0.0053) hopping compared to male rats. Overall, tickled rats produced more flat USVs in the one second before (X^2^_(1,63)_ = 86.02,* p *< 0.0001) and during (X^2^_(1,63)_ = 15.35,* p *< 0.0001) hopping compared to control rats, this was highest in tickled females before hopping (X^2^_(1,63)_ = 7.22,* p *= 0.0072). Within the control group, female rats emitted a higher mean number of flat 50 kHz USVs in the one second before hopping compared to males (t_ratio_ = 4.254,* p *= 0.0004) and the same was seen within the tickled group (t_ratio_ = 2.729,* p *= 0.0407) (Fig. 1). During hopping, there was no interaction of treatment and sex (X^2^_(1,63)_ = 0.31,* p *= 0.5768), as control female and male rats emitted similar numbers of flat USVs and the same was true for USV production during hopping in tickled female and male rats.

**Figure 2:**
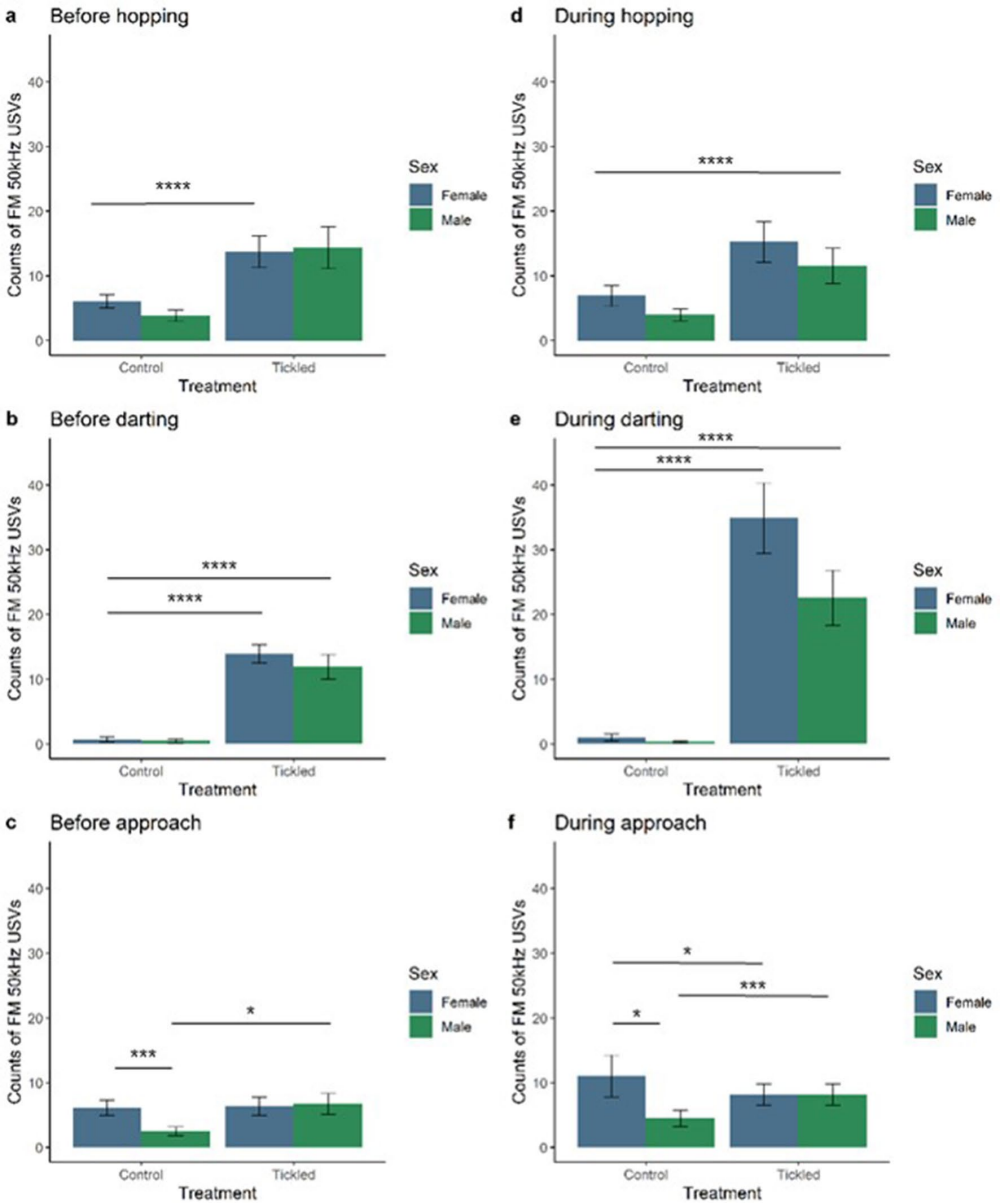
Frequency-modulated (FM) ultrasonic vocalisations (USVs) paired with play-related behaviours in tickled compared to control rats. Estimated marginal means  ± 95% CI of FM 50 kHz USVs produced in the one second before hopping (**a**), darting (**b**) and approach to the hand (**c**) ‘play-related’ behaviours, and FM 50 kHz USVs produced during hopping (**d**), darting (**e**) and approach to the hand (**f**) ‘play-related’ behaviours. Female (blue) and male rats (green). n = 16/group. *denotes *p* ≤ 0.05; **denotes *p* ≤ 0.01; *** denotes *p* ≤ 0.001; **** denotes *p* ≤ 0.0001 for significant pairwise interactions.

The interaction between treatment and sex had an overall effect on the number of FM USVs emitted in the one second before hopping (X^2^_(1,63)_ = 7.16,* p *= 0.0074) which was due to treatment as tickled female and male rats made more FMs before hopping than control female or male rats (Fig. 2). There was no significant interaction between treatment and sex on the number of FM USVs emitted during hopping (X^2^_(1,63)_ = 2.20,* p *= 0.1383). Treatment, but not sex, affected the pairing of FM 50 kHz USVs with hopping. Tickled rats, regardless of sex, made more FM USVs in the one second before (X^2^_(1,63)_ = 121.10,* p *< 0.0001) and during (X^2^_(1,63)_ = 98.71,* p *< 0.0001) hopping compared to control rats.

#### Darting

The interaction between treatment and sex did not have an overall effect on the number of flat USVs emitted in the one second before darting (X^2^_(1,63)_ = 3.47,* p *= 0.0624) and this is likely due to no differences between sexes in the control group (Fig. 1). During darting, there was no interaction between treatment and sex (X^2^_(1,63)_ = 3.22,* p *= 0.0730). Female rats had higher rates of flat USVs before (X^2^_(1,63)_ = 7.73,* p *= 0.0054) and during (X^2^_(1,63)_ = 6.62,* p *= 0.0101) darting compared to males. Tickled rats made more flat USVs before (X^2^_(1,63)_ = 182.04,* p *< 0.0001) and during darting (X^2^_(1,63)_ = 339.12,* p *< 0.0001) compared to control rats.

There was no effect of the interaction between treatment and sex on FM USVs before (X^2^_(1,63)_ = 0.06,* p *= 0.7938) or during darting (X^2^_(1,63)_ = 1.99,* p *= 0.1585; Fig. 2). As with hopping, tickled rats, regardless of sex made more FM USVs in the one second before (X^2^_(1,63)_ = 103.76,* p *< 0.0001) and during (X^2^_(1,63)_ = 284.87,* p *< 0.0001) darting compared to control rats and there was no overall effect of sex (Fig. 2).

#### Approaches to the hand

There was no effect of the interaction between treatment and sex on the number of flat 50 kHz USVs emitted before (X^2^_(1,63)_ = 0.33,* p *= 0.5643) or during approaches (X^2^_(1,63)_ = 1.36,* p *= 0.2434). There was a sex difference in flat 50 kHz USVs being paired with hand approaches (Fig. 1). Female rats emitted higher rates of flat USVs in the one second before (X^2^_(1,63)_ = 9.10,* p *= 0.0026) and during (X^2^_(1,63)_ = 7.92,* p *= 0.0049) approaches to the hand compared to males. Tickled rats made more flat USVs during approaches to the hand compared to control rats (X^2^_(1,63)_ = 5.27,* p *= 0.0217), but not before approaches (X^2^_(1,63)_ = 2.81,* p *= 0.0936.

The interaction between treatment and sex had an overall effect on the number of FM USVs emitted in the one second before approaching the hand (X^2^_(1,63)_ = 16.74,* p *< 0.0001; Fig. 2). Pairwise comparisons revealed that control male rats emitted lower mean numbers of FM 50 kHz USVs in the one second before approaching the hand compared to tickled males (t_ratio_ = − 5.37,* p *< 0.0001) and control females (t_ratio_ = 2.97,* p *= 0.0219). Similarly, the interaction between treatment and sex had an overall effect on the number of FM USVs emitted during hand approaches (X^2^_(1,63)_ = 23.25,* p *< 0.0001), with control males making fewer FM 50 kHz USVs during approaches than control females (t_ratio_ = 2.94,* p *= 0.0236), tickled females (t_ratio_ = 3.11,* p *= 0.0149) and tickled males (t_ratio_ =  − 4.08,* p *= 0.0008). Tickled rats, regardless of sex, made more FM USVs in the one second before (X^2^_(1,63)_ = 12.13,* p *= 0.0005) but not during (X^2^_(1,63)_ = 0.22,* p *= 0.6405) hand approaches as compared to control rats.

### USV production associated with non-play-related behaviours

#### Exploring

**Figure 3:**
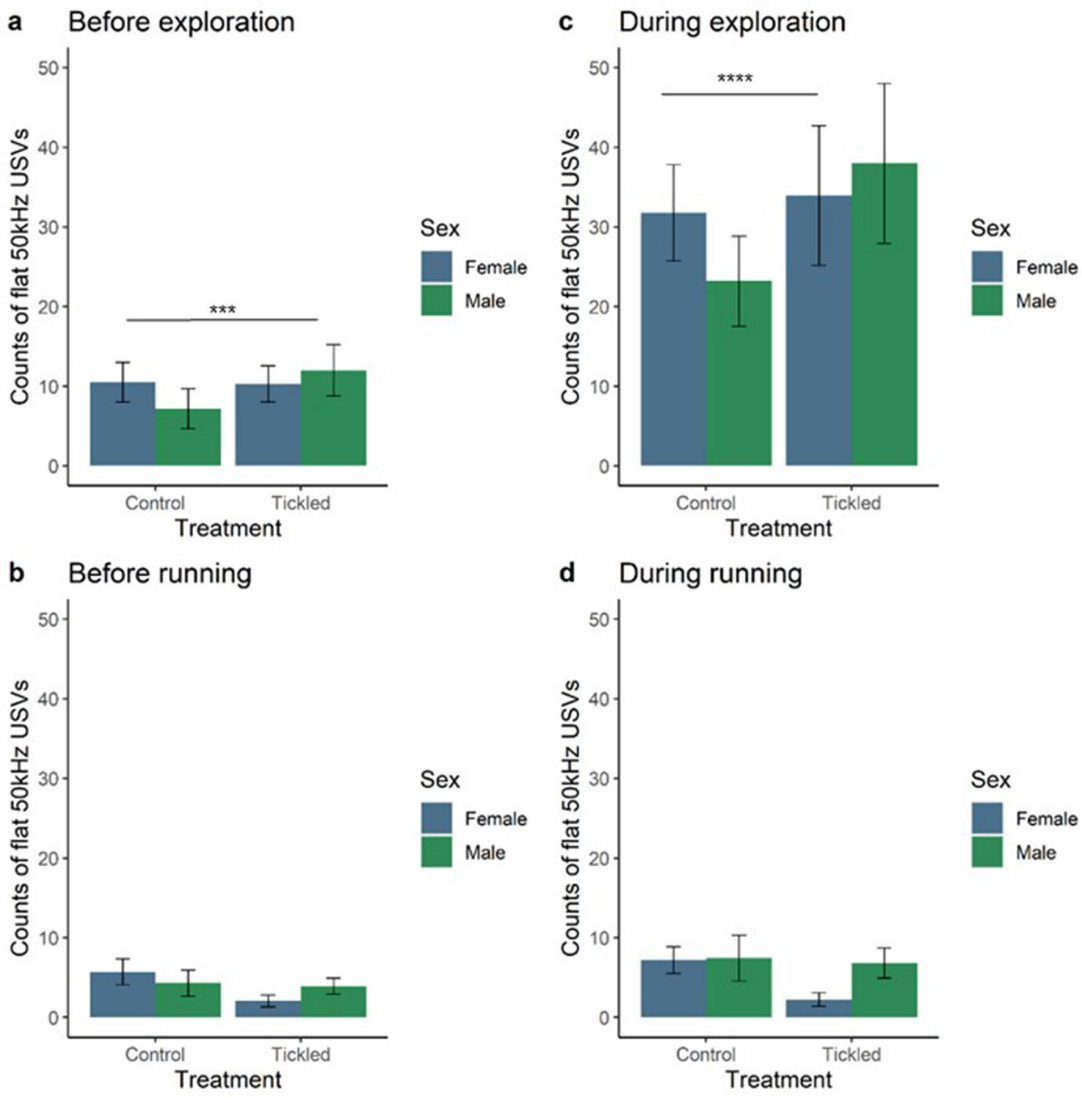
Flat ultrasonic vocalisations (USVs) paired with explorations, but not running, in tickled compared to control rats. Estimated marginal means  ±95% CI of flat 50 kHz USVs produced in the one second before exploration (**a**) and running (**b**), and flat 50 kHz USVs produced during exploration (**c**) and running (**d**) ‘non play-related’ behaviours. Female rats (blue) and male rats (green). n = 16/group. *denotes *p* ≤ 0.05; **denotes *p* ≤ 0.01; *** denotes *p* ≤ 0.001; **** denotes *p* ≤ 0.0001 for significant pairwise interactions.

The interaction between treatment and sex had an overall effect on the number of flat USVs emitted in the one second before (X^2^_(1,63)_ = 10.86,* p *= 0.0010) and during (X^2^_(1,63)_ = 22.41,* p *< 0.0001) exploring (Fig. 3). Pairwise comparisons revealed differences between control and tickled males, with control males emitting a lower mean number of flat 50 kHz USVs emitted before (t_ratio_ = − 4.35, *p* = 0.0003) and during (t_ratio_ = − 7.50, *p* < 0.0001) exploring than tickled males. In contrast to play-related behaviours, there was a treatment, but not a sex, effect on flat USVs being paired with exploration (Fig. 3). Irrespective of sex, tickled rats made more flat USVs before (X^2^_(1,63)_ = 8.062, *p* = 0.0045) and during exploration compared to control rats (X^2^_(1,63)_ = 34.98, *p* < 0.0001).

**Figure 4:**
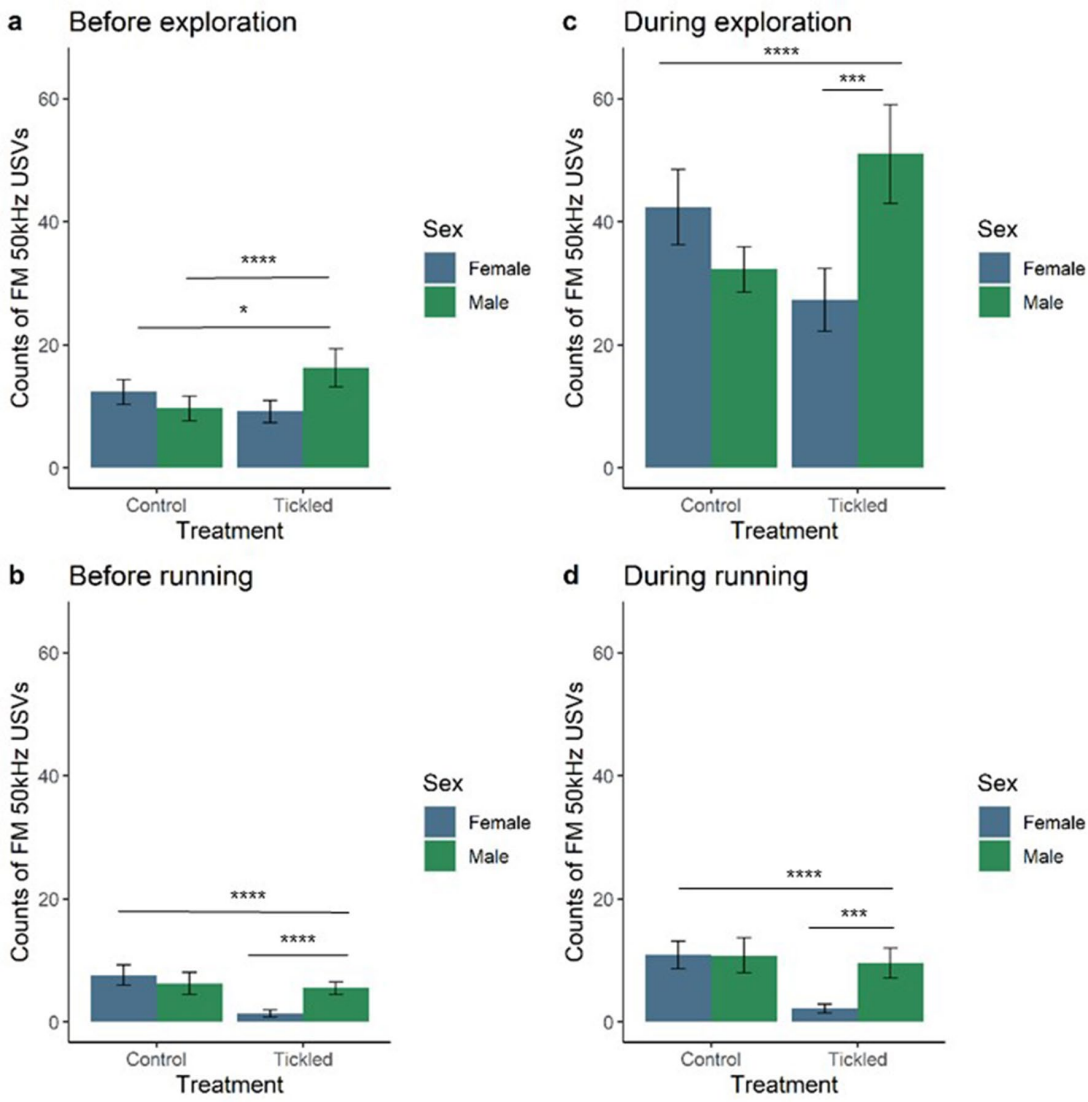
Frequency-modulated (FM) ultrasonic vocalisations (USVs) paired with explorations and running in tickled compared to control rats. Estimated marginal means ±  5% CI of FM 50 kHz USVs produced in the one second before exploration (**a**) and running (**b**) ‘non play-related’ behaviours, and FM 50 kHz USVs produced during exploration (**c**) and running (**d**) ‘non play-related’ behaviours. Female rats (blue) and male rats (green). n = 16/group. *denotes *p* ≤ 0.05; **denotes *p* ≤ 0.01; ***denotes *p* ≤ 0.001; **** denotes * p* ≤  0.0001 for significant pairwise interactions.

The interaction between treatment and sex had an overall effect on the number of FM USVs emitted in the one second before (X^2^_(1,63)_ = 29.99, *p* < 0.0001) and during (X^2^_(1,63)_ = 116.11, *p* < 0.0001) exploration (Fig. 4). Control females made more FM USVs before exploration than tickled females (t_ratio_ = 2.74, *p* = 0.0400), while tickled males had higher rates of FM USVs before exploration than control males (t_ratio_ = − 5.10, *p* < 0.0001). This was also observed during exploratory behaviour with control females making more FM 50 kHz USVs during exploration than tickled females (t_ratio_ = 7.15, *p* < 0.0001), and tickled males made more FMs than control males (t_ratio_ = − 8.14, *p* < 0.0001). Within the tickled groups, males emitted a higher mean number of FM 50 kHz USVs during exploration compared to females (t_ratio_ = − 4.09, *p* = 0.0008). In contrast to play-related behaviours, tickling did not affect FM USVs when paired with exploration. There was no main effect of treatment (before: X^2^_(1,63)_ = 3.47, *p* = 0.0624; during: X^2^_(1,63)_ = 1.36, *p* = 0.2434) or sex (before: X^2^_(1,63)_ = 0.55, *p* = 0.4589; during: X^2^_(1,63)_ = 1.44, *p* = 0.2295) on the number of FM 50 kHz USVs paired with exploration.

#### Running

The interaction between treatment and sex had no effect on flat 50 kHz USVs when paired with running (before: X^2^_(1,63)_ = 3.33, *p* = 0.0679; during: X^2^_(1,63)_ = 2.93, *p* = 0.0869; Fig. 3). Also, there was no effect of treatment (before: X^2^_(1,63)_ = 2.62, *p* = 0.1057; during: X^2^_(1,63)_ = 3.31, *p* = 0.0689) or sex (before: X^2^_(1,63)_ = 0.21, *p* = 0.6434; during: X^2^_(1,63)_ = 2.73, *p* = 0.0986; Fig. 3).

Tickled females had the lowest FM USV rate before and during running (Fig. 4). The interaction between treatment and sex had an overall effect on the number of FM USVs emitted in the one second before (X^2^_(1,63)_ = 32.66, *p* < 0.0001) and during (X^2^_(1,63)_ = 47.02, *p* < 0.0001) running and this may be explained by the low call rates of tickled females (Fig. 4). Control females (before: t_ratio_ = 7.34, *p* < 0.0001; during: t_ratio_ = 8.66, *p* < 0.0001), tickled males (before: t_ratio_ = − 4.59, *p* = 0.0001; during: t_ratio_ = − 4.56, *p* = 0.0002) and control males (before: t_ratio_ = − 5.03, *p* < 0.0001); during: t_ratio_ = − 4.94, *p* < 0.0001) emitted a higher mean number of FM 50 kHz USVs before and during running compared to tickled females. Control rats made more FM USVs in the one second before (X^2^_(1,63)_ = 21.97, *p* < 0.0001) and during running compared to tickled rats (X^2^_(1,63)_ = 29.14, *p* < 0.0001), regardless of sex.

## Discussion

To our knowledge, this is the first study to show that there is a sex difference in flat 50 kHz USVs being emitted in relation to certain tickle-induced behaviours. Female rats paired more flat 50 kHz USVs than males with hopping and darting solitary play behaviours. The estimated marginal means suggest a strong trend for tickled female rats to pair more flat USVs with hopping and darting than tickled males and control rats. Females, irrespective of treatment, paired flat USVs with approaches to the hand significantly more than males. In contrast, tickled rats of both sexes paired FM 50 kHz USVs with hopping, darting and approach behaviours significantly more than control rats. This suggests that flat USVs are being used during these behaviours differently in female rats, while tickling induces higher FM USV call rates with hopping, darting and approaches, irrespective of sex. It should be noted that the behavioural responses to tickling observed in the present study may in part be influenced by the use of a reverse light cycle; the phase of light cycle has been shown to affect the response of female and male rats to chronic stress, where chronic stress lead to an increase in anxiety-like behaviours in the dark, but not light, phase^67^. To the best of our knowledge, there is no study to date directly investigating the effect of light phase on the response to tickling.

It is clear from these data that there are patterns in the association of USV subtypes and behaviours in juvenile Wistar rats, which is similar to the findings from Burke et al.^22^ where they found that calls do not appear to be produced randomly but are linked to certain play behaviours in male Long-Evans rats. Takahashi et al.^28^ similarly found that subtypes of USVs correspond with fighting, feeding and locomotive behaviours. In other species, ultrasonic vocalisations have a role in coordinating behaviours. For example, USVs are thought to maintain social cohesion in slow loris’ (*Nycticebus javanicus*)^29^, kin recognition in grey mouse lemurs (*Microcebus murinus*)^30^ and social communication in the common (Microtus arvalis), bank (*Myodes glareolus*) and field (*Microtus agrestis*) vole species^31^. Similarly, USVs are thought to function to coordinate playful actions between rats [e.g.^22,32^]. As vocalisations during tickling may play a similar role, it could be that rats are using calls for specific functions during tickling. The current study adds to the findings of other studies which have mainly focussed on male Long-Evans rats^22^ by showing coupling of UVSs to behaviours also in female and male Wistar rats.

Control rats called infrequently both before and during play-related hopping and darting behaviours, while tickling induced higher call rates both before and during play-related behaviours in both sexes suggesting that USVs are linked to play behaviours elicited during tickling. As there was an association of FM both before and during play-related behaviours it suggests that FM are used by both sexes as signals of affective state (induced by both conspecific and heterospecific playful interactions). This provides evidence that FM may act as an indicator of positive affect, or even to enhance positive affect^19,33^.

### Calls paired with play-related behaviours

The findings from the present study suggest that female and male juvenile Wistar rats may differentially use FM and flat calls as an affective signal during tickling. Tickling resulted in higher numbers of flat 50 kHz USVs produced one second before hopping and darting behaviours, and higher numbers of FM USVs before hops, darts and approaches. Female rats emitted more flat, but not FM, 50 kHz calls than males before hopping, darting and approaching. Tickled rats of both sexes make more FM calls before play-related, versus non-play-related, behaviours. This supports previous findings for 50 kHz USVs, particularly of the FM subtype, being a play signal. FM have been found to be important for playful encounters and they may facilitate playful contact^19^. Similarly, rats are found to emit calls immediately before making playful contact in conspecific social play^34^, and pre-contact calls are emitted by both playmates during conspecific social play^35^.

The present study provides evidence for the first time that tickled female rats produce more flat, rather than FM, 50 kHz USVs in response to tickling. Tickled female rats emitted more flat USVs in relation to hopping, darting and hand approaches than other groups. Based on the few studies to use both sexes in tickle experiments, there are inconsistent findings on whether female or male rats vocalise more in response to tickling [e.g.^12–14,36^]. There is evidence that female rats from three outbred stocks (Wistar, Long-Evans and Sprague–Dawley) produce multiple types of 50 kHz USVs in response to being tickled^37^. There has been a tendency in previous tickling studies to focus on FM 50 kHz USVs [e.g.^1,18^]. Female rats produce flat USVs abundantly during mating^38^ which is indicative of flat 50 kHz USVs having a sex-specific communicatory role for female rats. It is possible that females were producing more flat 50 kHz USVs than males due to the sex of the experimenter; this has been previously discussed by Lafollette et al.^15^ who, similarly to the present study, found that female rats called more than male rats and a female experimenter carried out the tickling. This is of relevance, since it has been shown that the sex of the experimenter influences the behavioural response to pain in rats^66^.

Tickled rats made more flat calls during play-related behaviours than control rats, specifically tickled females emitting more flat calls than other groups. Burke et al.^22^ also found that flat calls were associated with conspecific play behaviours such as active wrestling between two juvenile male rats, and were not associated with passive contact. Flat calls have been postulated to be used as cues of dominance and submission between playing juvenile male rats^22^ and this communication may be used to avoid escalation of play fighting to aggression^39^. It is plausible that flat USVs may be used by each sex differently. For example, in males flat USVs may be used to establish dominance^22^. Further investigation is needed to address the purpose of flat USVs for females.

Female and male tickled rats made more FM during the play related behaviours such as hopping and darting. USVs have been previously linked to hopping and darting in the context of sexual behaviours^40^, particularly for females. FM USVs have been positively correlated with conspecific social play and heterospecific play in female and male Long-Evans rats^18^, and calls that had FM elements have been associated with play behaviours, such as nape contacts, chasing and wrestling^22^. In a number of studies, FM USVs have been related to reward and positive affect [e.g.^2,5,18,41^]. Therefore, this may indicate that hopping and darting behaviours were associated with the reward of tickling in the present study. In the present study, FM seemed to be emitted just before but not during approaching the hand. Burke et al.^22^ found that FM were also associated with approaches that were followed by a playful contact, but not when followed by a non-playful contact. Hand approach behaviour is often used as an indicator of whether tickling is a rewarding experience^2^, however, there are inconsistent effects of tickling on measures of approach have been reported in previous studies^8,15,27^. Therefore, approach behaviours may not be as reliable measure of the reward of tickling as FM USVs.

### Calls paired with non-play-related behaviours

Greater numbers of flat 50 kHz USVs were made before exploration by tickled compared to control rats, while control rats emitted more FM before running. Manduca et al.^42^ found that both male Wistar and Sprague–Dawley rats emitted similar numbers of USVs during social and non-social behaviours, such as cage exploration and self-grooming. This is similar to our finding that tickled rats made more FM and flats during play-related behaviours and also made more flats during exploration, although there was no association between flat 50 kHz USVs and running. LaPlagne and Costa^43^ also found that 50 kHz USVs do not appear to be just a by-product of vigorous movement, although USVs were associated with locomotion. Burke et al.^24^ found that running was associated with FM calls in juvenile male Wistar rats and flat calls had less of an association. In the same study flat and FM USVs did not have a strong association with exploration behaviours, which is consistent with this present study where tickled females made very few FM or flat USVs before or during running. This is similar to the finding that male rats were less likely to call during non-social behaviours, and calls were associated more strongly with playful behaviours^22^.

## Conclusions

We conclude that juvenile Wistar rats couple reward-associated FM 50 kHz USVs with play-related behaviours of hopping, darting and approaching, and importantly that this finding is consistent between females and males. Tickling has a substantial effect on USVs associated with play-related behaviours. A key finding is that female rats produce more 50 kHz USV flats in association with play-related behaviours compared to males. As flat and FM USVs are thought to act in a communication role during play, this indicates that there are sex differences in the use of different USV subtypes and this is an important to take into consideration when studying rat social and playful handling behaviour.

## Methods

### Subjects

Across two replicates, 64 juvenile Wistar rats (Replicate 1: Females 41.0–69.1 g, Males 48.6–75.5 g; Replicate 2: Females 39.1–62.4 g, Males 42.7–64.3 g) were sampled (32 per replicate; Charles River, Kent, UK). Each replicate was split evenly between males and females. Rats arrived at the Roslin Institute Bioscience and Veterinary Services facility at 23–24 days of age. The rats were derived from four different litters: four female and four male rats from the same litter were used (four litters in total). Treatment (Control or Tickled) was randomly assigned to each rat, balancing for body weight and littermates so that average weights for each treatment group and for each sex were as balanced as possible. There was an equal number of animals from each litter in both treatment groups.

Rats were housed in same-sex pairs, with each cage containing a tickled and control rat (the tickled rats were marked with a black mark on their tail in marker pen, control rats had no mark, treatment was pseudo-randomly assigned balancing for body weight^44^ and litter (no littermates were housed in the same cage)). Standard clear plastic cages (46 × 25 × 21 cm) with a wire lid were used. Each cage contained aspen chip bedding, one shredded paper nest, one aspen chew stick (Nepco, Warrensburgh, USA). Food (14% protein rodent maintenance diet, Envigo, UK) and water were available ad libitum and the room temperature, humidity and light intensity was held stable at 18–23 °C, 40–60% and 25 lx respectively. The cages were pseudorandomly arranged in a cage rack to account for differing lux levels through the height of the rack and balanced for sex and litter. The rats were held on a reversed 12-h light/12-h light dark cycle (lights on: 00:00, lights off: 12:00) and were tested in the tickling test arena in the dark phase. Body weight (g) was recorded daily following testing between 16:00 and 18:00 in the dark phase. The rats were checked daily (by laboratory personnel at 08:00, during the light phase) and nitrile gloves were worn when handling the animals. To minimise handling stress rats were picked up gently by holding them behind their forelegs and then cupping them with both hands. Following arrival rat body weight (g) was taken daily and rats were acclimatised to their new surroundings for five days before they were habituated to the tickling test arena (a Perspex open box, 60 (length) × 60 (width) × 25 (height) cm that was lined with LabMat; LabLogic Systems Ltd., England).

### Experimental design

Rat sample size was determined using a power equation using variance and mean values from previous data^27^ (calculated sample size: 16 per group). The order in which the cages and cage mates were tested each day was pseudo-randomised to account for time of day, sex, treatment and lux levels of the cages in the cage rack.

All testing (weighing, habituation and tickling) was carried out in the home cage room. The area used for all testing was enclosed by a thick, plastic curtain, at the opposite end of the room to the cage rack. Testing was carried out in the dark phase under red light for the experimenter to see; only one experimenter (female) carried out the testing and was the only person present during testing. The rats were brought to a bench in the enclosed area used for testing in their home cage. One rat was tested while the second cage mate remained in the home cage. The home cage was placed on the other side of the curtain from the testing apparatus so that no USVs were detected by the rat in the home cage (the curtain blocked USV transmission, measured using the ultrasonic microphone).

Animals were given a five-day habituation period during which they were placed alone in the centre of the testing arena for a total of five minutes per day. An immobile right hand (wearing a nitrile glove covered with a white cotton glove) was placed in the arena to habituate the rats to the glove and the researcher.

After the habituation phase, on day six, the rats began ten days of behavioural testing. Each animal was tested in the arena for two minutes per day for ten days. One cage mate (randomised order) was taken from the home cage and placed in the test arena. The rat was tested for two minutes, timed with a stopwatch and both video and sound recordings of the behaviour were recorded digitally using a video camera placed above the testing arena (Panasonic HD HC-V10) and an ultrasound microphone suspended about 30 cm above the testing arena (Pettersson M500-384 USB Ultrasound microphone, PetterssonElectronik, Sweden). The arena was cleaned with 70% ethanol gel and allowed to dry between the testing of each rat and testing was carried out in the first three hours of the dark phase.

For the tickled group, rats were placed in the arena, and a hand (wearing a white cotton glove) was placed motionless on one wall of the arena (the wall position and placement of the hand was randomised each time) for the first 15 s of testing. Following these 15 s of release (i.e. where the experimenter’s hand was motionless on the side of the arena and the rat received no contact with the hand), the rat was tickled for 15 s by making rapid finger movements on the nape of the neck. If the rat turned its body around to rear up at the hand or rolled onto its back during tickling, it was also tickled on its ventral side; the rats were not manually flipped and pinned as described elsewhere^1^. This has been described as ‘playful handling’ in^9^. The 15-s bouts of tickling and release were alternated during the two minutes of testing. For the control group, rats were placed in the arena for two minutes, with a hand (wearing a white cotton glove) resting motionless on one wall of the arena (the wall position and placement of the hand was randomised each time). If the rat reared at the hand, the hand was gently moved away to one side and then replaced back in position. Following testing, the rat was gently picked up (as described previously) and body weight was measured before it was returned to its home cage. The other cage mate was then removed (following cleaning of the arena described above) and placed in the arena and the behavioural testing was repeated. The cotton gloves were only worn by the hand in the testing arena during testing and each rat was exposed to its own individual cotton glove to avoid any potential olfactory bias.

All animal work was carried out in accordance with the UK Animals (Scientific Procedures) Act 1986 following ethical approval by the Roslin Institute’s Animal Welfare and Ethical Review Body (AWERB study number B026), and carried out in the Roslin Institute’s Bioscience and Veterinary Services Facility. The authors complied with the ARRIVE guidelines.

### Measurements

#### Ultrasonic vocalisation analysis

An ultrasound microphone and Audacity software (Version 2.1.3, Pennsylvania, United States of America) were used to record the spectrograms of USVs. The number of FM and flat 50 kHz USV events emitted during the two-minute testing phase on the final day of testing for each animal were counted manually from spectrograms produced in Ultravox 14 (Noldus Information Technology, Wageningen, Netherlands; Tracksys Ltd, UK). Call events were visually categorised using call parameters for FM and flat calls outlined by Wright et al.^17^ (see Table 1).

**Table 1:** Ethogram used to carry out the behavioural analysis of the rats response to tickling. The following table defines the list of rat behaviours quantified with their description and reference to previously published studies.

Behaviour	Definition	References
Exploration	Sniffing directed at the environment, either when still or during slow walking, including rearing behaviours. Each bout of sniffing (with or without rearing) was measured- a bout was determined as > 1 s participating in the behaviour	^12,50^
Run	Locomotion which is not locomotor play, so does not include scampering, hopping or darting. Slower locomotion than darting, at least one paw is on the floor at any given time, not directed at or in response to the hand, usually in one direction. A bout was determined as > 1 s participating in the behaviour	^48^
Hop	‘Joy-leaps’/ ‘jerk-jumps’. One hop was determined as when all four feet left the floor	^26,45,46^
Dart	Rapid darting movements, locomotion with frequent changes in direction. One dart was determined as a rapid locomotory movement in the absence of sniffing, lasting > 1 s, usually in a circular motion returning to the hand	^26,46–48^
Approach	Forward motion, directed movements including rears directed at the hand of the experimenter. One approach was determined as a forward locomotor movement directed towards the hand ending in the rat touching the hand with the nose	^49^
Flat 50 kHz USV	Calls that have a nearly constant frequency in the 30–90 kHz frequency range with a mean slope between − 0.2 and 0.2 kHz/ms, those include calls in the *flat* USV category according to the classification in^17^. Any *flat* calls combined with a *trill* call as in *flat-trill combinations, and composite* USV categories in^17^ were not included in the analysis	^17^
FM 50 kHz USV	Calls in the 30–90 kHz frequency range, containing short (< 15 ms) sinusoidal oscillatory motifs, those include calls in *trill, complex, multistep, trill with jumps* according to the classification in^17^*.* Any *trill* calls combined with a *flat* call as in *flat-trill combinations, and composite* USV categories in^17^ were not included in the analysis	^17^

#### Testing arena behaviour

Video camera (Panasonic HD HC-V10) footage was obtained during the two-minute testing phase on each day of testing. Test arena behaviour during the two minute testing phase on the final day was analysed in Observer 15 (Noldus Information Technology, Wageningen, Netherlands; Tracksys Ltd, UK) by the experimenter who was blinded to sex but not to treatment due to being able to see whether individual rats were tickled or not in the digital recording. The total number of hops, darts, approaches, exploration and runs observed during the 2-min testing phase were scored in Observer 15 using the ethogram shown in Table 1. Hops, darts and approaches were assigned as being ‘play related’ behaviours because they are observed during conspecific and heterospecific play in rats^26,27,45–49^. Exploration and runs were assigned as ‘non-play related’ behaviours because they are described as locomotion which is not associated with playful behaviours such as scampers or jumps [e.g.^48,50^]. These behaviours were used to compare the number of USVs produced during or before non-play related locomotion and during or one second before locomotor play behaviours. A one second duration before a behaviour was selected to allow for human error in coding behaviour times^22^. This was in addition to whether there was emission of USVs in the anticipation of play-related behaviours^3^ given that there may be association of calls with a behaviour up to 600 ms before the call is emitted^19^.

#### Synchronising USVs and observed behaviours

USV data from the final day were imported into Observer 15 from Ultravox 14. The video footage and sound files from Ultravox were then played concurrently and the behaviours scored using the ethogram (Table 1) which was written in Observer 15. This generated a file for each animal where the behaviours and USVs can be temporally compared: the USVs produced by the rat during or in the one second leading up to a behaviour of interest were counted. Using Observer 15, data profiles were built for each group (tickled female, tickled male, control female, control male) and the number of flat or FM 50 kHz USVs made during, and one second before any of the scored behaviours were counted.

#### Statistical analysis

Statistical analysis was carried out in R Studio and R (v 4.0.3, The R Foundation for Statistical Computing Platform (2020)). Model adequacy was verified by examination of residuals^51^ via the DHARMa package^52^. Generalised linear mixed models (GLMMs) using the glmmTMB package^53^ were used to compare frequencies of flat and FM 50 kHz USVs emitted before or during observed locomotory behaviours within allocated treatments. Dependent on model fitting and overdispersion, family links were set to either poisson or negative binomial distributed errors (‘nbinom2’ in the MASS package)^54^ with default transformations. All models included batch and cage as random effects and were nested (batch followed by cage). This was done to account for the variation from the non-independence of rats from the same cage and potential batch effects. All models included both sex and treatment as fixed effects, as well as the interaction between them, with effects reported through ANOVA comparisons via the car package^55^ to compare the differences between group means rather than the linear relationships between variables. Pairwise comparisons were identified and reported using the emmeans package^56^, with statistical significance based on *p* < 0.05 threshold level and adjusted for multiple comparisons using the Tukey method^56^. All graphs were generated in R Studio. The data in the graphs are presented as the estimated marginal means ± CI.

## Data Availability

The datasets generated during and/or analysed during the current study are available from the corresponding author on reasonable request.
